# Surface Plasmon Resonator Using High Sensitive Resonance Telecommunication Wavelengths for DNA Sensors of *Mycobacterium Tuberculosis* with Thiol-Modified Probes

**DOI:** 10.3390/s150100331

**Published:** 2014-12-25

**Authors:** Shih-Hsiang Hsu, Shao-Chiang Hung, Yu-Kun Chen, Zhi-Hao Jian

**Affiliations:** Department of Electronic Engineering, National Taiwan University of Science and Technology, No. 43, Sec. 4, Keelung Rd., Taipei 10607, Taiwan; E-Mails: leo12392000@gmail.com (S.-C.H.); chen10284@gmail.com (Y.-K.C.); b98530121@ntou.edu.tw (Z.-H.J.)

**Keywords:** *Mycobacterium tuberculosis*, surface plasmon resonance, immobilization, wavelength modulation

## Abstract

Various analytes can be verified by surface plasmon resonance, thus continuous improvement of this sensing technology is crucial for better sensing selection and higher sensitivity. The SPR sensitivity on the wavelength modulation is enhanced with increasing wavelengths. The telecommunication wavelength range was then utilized to detect *Mycobacterium tuberculosis* (*MTB*) deoxyribonucleic acid (DNA) under two situations, without immobilization and with 5′-thiol end labeled IS6100 DNA probes, for SPR sensitivity comparison. The experimental data demonstrated that the SPR sensitivity increased more than 13 times with the wavelength modulation after immobilization. Since the operating wavelength accuracy of a tunable laser source can be controlled within 0.001 nm, the sensitivity and resolution on immobilized *MTB* DNA were determined as 1.04 nm/(μg/mL) and 0.9 ng/mL, respectively.

## 1. Introduction

Almost three million people in the world die every year from tuberculosis (TB), which is an infectious disease only caused by pathogens. The majority of deaths from TB could be prevented through early diagnosis and treatment. However, in the areas of prevailing TB, a combination of sputum smear and culture is typically utilized for checking the presence of *Mycobacterium tuberculosis (MTB)*. Unfortunately the bacterial growth step can take 4–6 weeks, and earlier diagnosis is crucial in effective TB control.

In recent years, the immune response marker to MTB was typically utilized as an infection examination [1]. However, the biomarkers and detection reagents used in this case are protein-related antibodies or antigens as reagents, which are unstable after long exposure to a room temperature environment and are also easily degraded due to temperature variations. On the other hand, the preservation and stability of nucleic acids are reliable. Due to the lack of robustness of the antigen/antibody based tests, recent work has been directed towards using DeoxyriboNucleic Acid (DNA) detection methods. Therefore *MTB* DNA is utilized as the detection reagent or biomarker [2–6].

Specific DNA sequence amplification using polymerase chain reaction (PCR) is a sensitive method for mycobacterial detection [7,8]. However, false-positive results could be generated due to amplified DNA contamination from the PCR laboratory. Moreover, this technique requires expensive reagents and testing devices and well-trained personnel [9], making it necessary to search for more efficient diagnostic methods. Surface plasmon resonance (SPR) sensors have made significant progress in both the technical and application aspects, mainly because of their label-free features and instant detection capabilities, which includes the detection of bacteria, viruses, toxins, allergens, and biomedical analytes, besides environmental pollutants [10].

SPR is a physical phenomenon that happens between the interface of metal and dielectric materials. This feature allows for real time, high sensitivity and label-free detection in biosensing applications, therefore this method has been extensively utilized in bio-detection and immunochemistry for its efficiency in analyzing the refractive index of detected materials. The SPR performance is heavily related to the surface plasmon effective index, which is severely affected by the immobilization between the analyte and the Au metal layer, therefore numerous strategies for reagent immobilization were developed for different types of recognition elements, including the proteins, peptides, DNA and more complex natural products [11].

In the current study, the analyte and its corresponding binding reaction with the receptor could be instantly detected using this method. A SPR sensor for MTB DNA was developed based on the stable and repeatable tunable laser wavelength modulation of optical fiber communications. Here, the immobilization with 5′-thiol end labeled DNA probes was applied to enhance the SPR sensitivity and resolution compared with sensing without probes.

## 2. Design

Because infrared light has longer penetration depth of the surface plasmon than visible light, SPR was utilized to study living cells and analyses of cholesterol penetration into plasma membranes and transferrin-induced clathrin-mediated endocytosis were demonstrated [12,13]. The relatively large penetration depth of the surface plasmon into a dielectric medium, a few microns in the infrared range, is of the order of the cell height and beneficial in studying cell cultures. The long-wavelength surface plasmon penetration may even sense the whole cell volume. SPR penetration depth and propagation length measured around 1 μm and 30 μm, respectively, for the interfaces of Au/water and ZnS/Au/water at a 1550-nm wavelength [12].

Moreover, the SPR sensor sensitivity with wavelength modulation is enhanced with an increasing wavelength, as shown in the following [11]:

(1) $$\left(\frac{\delta \lambda_{r}}{\delta n_{ef}}\right)=\frac{1}{\left|\frac{dn_{p}}{d\lambda }\frac{n_{ef}}{n_{p}}-\frac{dn_{ef}}{d\lambda }\right|}$$

where *λ_r_* is the resonance wavelength. $\frac{dn_{ef}}{d\lambda }$ and $\frac{dn_{p}}{d\lambda }$ are the dispersion of the effective index of the surface plasmon and coupling prism, respectively.

In Equation (1), the sensor sensitivity is primarily determined by the second term in the denominator, $\frac{dn_{ef}}{d\lambda }$ [11]. Since the surface plasmon effective index decreases with longer wavelengths, its dispersion has a negative value and increases with the longer wavelength [11]. The sensor sensitivity increases with increasing wavelengths due to the smaller absolute dispersion tendency of the surface plasmon effective index.

## 3. Experiments

### 3.1. Reagents and Oligonucleotides

MTB DNA was prepared by constructing five representative MTB genes (IS6110, 16S ribosomal RNA, 85B, Rv3130c and Rv3133c) into a pUC57 vector. The MTB DNA length was 3133 base pairs. The recombinant *MTB* plasmids were purified using the Qiagen plasmid purification kit (Qiagen, Valencia, CA, USA) according to the manufacturer's instructions and quantitated using NanoDrop™ 2000c (Thermal Fisher Scientific, Wilmington, DE, USA). Used chemicals were analytical reagent grade. Distilled water (18.2 MΩ) was used throughout these experiments.

### 3.2. Apparatus

The prism was sent into the e-beam evaporator for Cr and Au deposition, at thicknesses of 3 nm and 30 nm, respectively. Cr metal was utilized as an adhesive layer. The prism surface was adjusted to an appropriate angle in the deposition chamber so that the metal coverage could be uniform.

**Figure 1.**
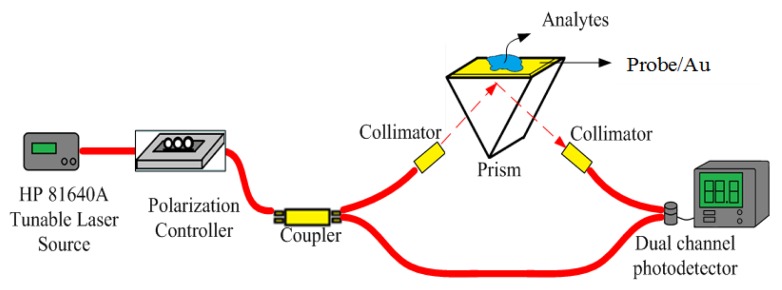
The fiber based testing setup for SPR wavelength modulation.

Since the tunable laser source (TLS) wavelength accuracy from the HP 81640A instrument could reach 0.001 nm, wavelength modulation was utilized to improve the sensitivity and resolution of the SPR characterization. After the incidence angle was fixed at around 61.9°, which is the resonance angle from angle modulated SPR on deionized (DI) water at a 1550-nm operating wavelength, the telecommunication wavelengths were executed using the TLS and modulated with 0.001-nm wavelength increases crossing the resonance wavelength. The coupling strength between the analytes and surface plasmon could be observed to change by adjusting the wavelengths. To ensure the experimental accuracy couplers should be used to monitor the input and output power using a dual-channel power meter, as shown in Figure 1. Due to the limited supply of MTB DNA only 1, 5, 6 and 9 μg/mL concentrations were used to demonstrate the wavelength modulation without immobilization and then implemented with 5′-thiol end DNA probes for the comparison of sensitivity and resolution.

### 3.3. Immobilization and Hybridization

The thiol labeled IS6110 DNA (5′ SH-CTAAC CGGCT GTGGG TAG) prepared in autoclaved deionized (DI) water was immobilized onto a pre-cleaned Au surface for 8500 s at 25 °C. The molecules were then successfully bound as follows [2]: prior to the immobilization of 5′-thiol end labeled DNA, Au plates were treated with piranha solution (7 H_2_SO_4_:3 H_2_O_2_) followed by DI water rinsing and subsequent ultrasonication method in absolute ethanol for 2 min together with consecutive DI water rinsing again. The 5′-thiol end DNA solution with 20 μM was prepared in DI water and immobilized onto the pre-cleaned Au surface for 8500 s at 25 °C. The Au surface was then blocked by incubating the desired Au surface in 1mM 6-mercapto-l-hexanol (MCH) solution for 2 h to avoid any non-specific binding. Finally the DNA immobilized bioelectrode was formed and washed thoroughly with DI water to remove any unbound probe. The isolated genomic MTB DNA of 10 μg/mL concentration was sonicated for 6 min in a 75% duty cycle with 30-second signal for DNA denaturation and then followed by 95 °C heating for 5 min.

## 4. Results and Discussion

Under the prism-coupled SPR sensing conditions, the reflected resonant optical power and wavelength are related to the detected analyte and Au film on the prism. The reason to use the resonance wavelength as the MTB DNA sensing indication instead of reflected resonant optical power was mainly for its highly sensitive performance.

**Figure 2.**
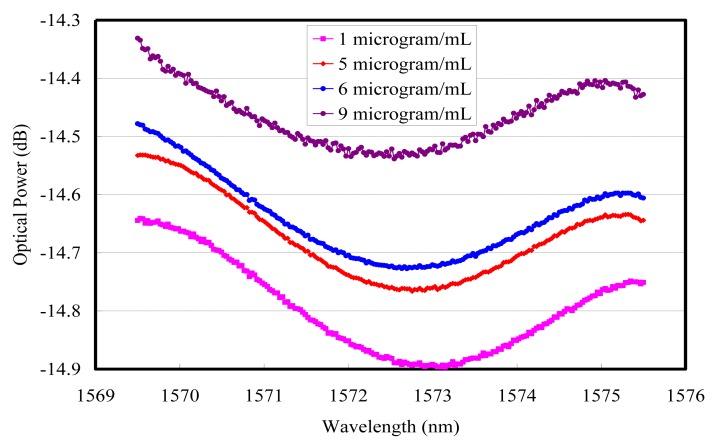
SPR wavelength modulation for different *MTB* DNA concentrations without immobilization.

**Figure 3.**
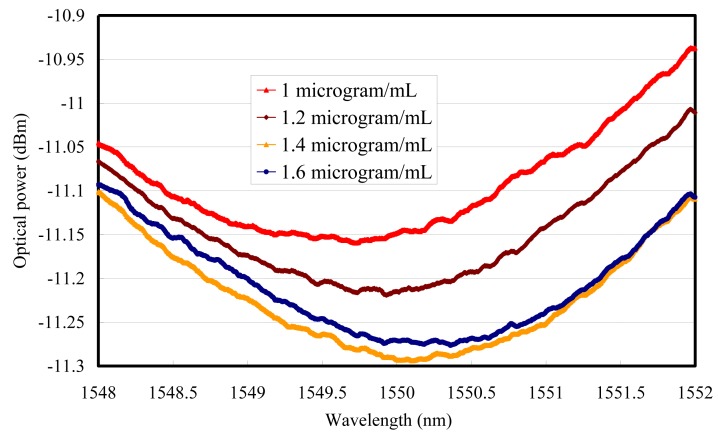
SPR wavelength modulation for *MTB* DNA concentrations under 5′-thiol end DNA probing.

The resonance wavelength moved to the longer side when the MTB DNA concentration was from 9 μg/mL to 1 μg/mL, as shown in Figure 2. Then the SPR immobilization experiments for *MTB* DNA detection were executed by DNA probes. After applying 5′-thiol end DNA, the resonance wavelength moved to the shorter side when the MTB DNA concentration was from 1.6 μg/mL to 1 μg/mL, as shown in Figure 3. There are multiple observed interferences in Figures 2 and 3, which appear between Au and analytes due to the high coherence of TLS [14]. The sensing limit is from the unstabilized polarization state of the input SMF-28 fiber based collimator combined with the optical path noise from amplitude modulation [15]. Therefore the transverse-magnetic (TM) polarized optical power from the tunable laser source with a telecommunication wavelength range cannot be kept constant. A tunable laser source with the polarization maintained fiber would be useful to improve the sensing limit. With proper design of a detection scheme, phase noises can be lower compared to amplitude ones, which results in a much better signal-to-noise ratio [15].

The resonance locations from the wavelength modulation varied with different MTB DNA concentrations. We need to notice that the resonance wavelength from various MTB DNA concentrations without immobilization was different from in the situation of 5′-thiol end DNA. The deviation was coming from the fixed angle before SPR wavelength modulation. Due to our stepper motor stage uncertainty, less than 1-degree angle accuracy would cause the resonance wavelength shifting. In non-immobilization, the lower MTB DNA concentrations are associated with longer resonance wavelengths. On the other hand the wavelength modulation on immobilized MTB DNA demonstrated that the resonance wavelengths would shift to the longer side when the MTB DNA concentration increased.

**Figure 4.**
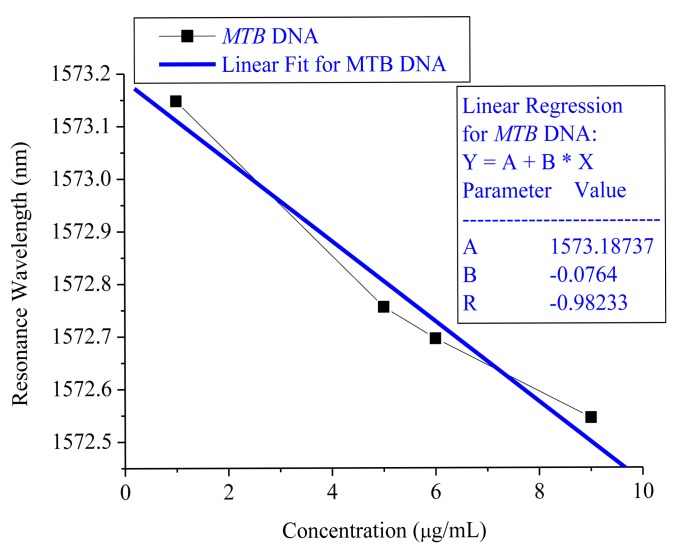
The MTB DNA sensor sensitivity in non-immobilization conditions.

The detected MTB DNA concentrations were categorized as 9, 6, 5 and 1 μg/mL. The wavelength modulation showed that the resonance wavelength difference was 0.603 nm from 9 μg/mL to 1 μg/mL under non-immobilization conditions and 0.635 nm from 1 μg/mL to 1.6 μg/mL under immobilized DNA conditions. The sensitivity from the linear slope without immobilization was −0.0764 nm/(μg/mL) and its linear regression R was 0.98233, as shown in Figure 4. 0.001 nm wavelength accuracy was secured from the HP 81640A TLS and the MTB DNA resolution without probing was 0.01 μg/mL.

As for the correlation between the resonant wavelength and reflected resonant optical power in non-immobilization conditions, the resonance wavelength measured −0.0764 nm/(μg/mL) and 0.01 μg/mL, respectively, for sensitivity and resolution when the wavelength accuracy of the HP 81640A tunable laser source was 0.001 nm. In Figure 2, the reflected resonant optical power gave −0.046 dB/(μg/mL) as sensitivity and the resolution was 0.217 μg/mL, similar to the values of a previous publication [6], when 0.01 dB accuracy of the optical power meter was achievable. The sensitivity from the resonance wavelength is 10 times higher than the reflected resonant optical power.

**Figure 5.**
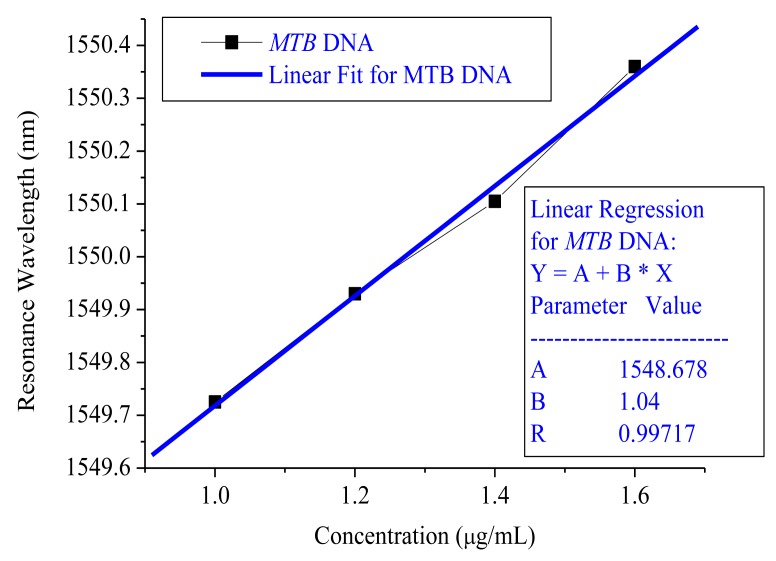
The MTB DNA sensor sensitivity under 5′-thiol end DNA probing conditions.

In Figure 5, the DNA sensor sensitivity for immobilized MTB is 1.04 nm/(μg/mL) and its linear regression R was 0.99717. Like the non-immobilization situation, the resolution could be derived as 0.6 ng/mL.

The sensitivity was improved from −0.0764 to 1.04 nm/(μg/mL), or more than 13 times, after applying 5′-thiol end DNA probing. The sensitivity difference between non-immobilization and immobilization for MTB DNA showed that the surface plasmon propagation constant was probing-related. We can also say that the various analytes alter the coupling condition between a light wave and the surface plasmon, which can be observed as a change in one of the characteristics of the optical wave interacting with the surface plasmon [11].

The sensitivity without immobilization showed a negative sign and represented the red-shift resonance wavelength for lower MTB DNA concentrations. The positive sensitivity in MTB DNA after probing showed a red-shift resonance wavelength for higher MTB DNA concentrations. We concluded that the propagation constant of the surface plasmon would decrease and increase, respectively, with non-immobilization and immobilization of via 5′-thiol end DNA, for higher MTB DNA concentrations.

In non-immobilization conditions, lower MTB DNA concentrations were associated with longer resonance wavelengths [16], which could be described qualitatively as lower reflective optical power due to less MTB DNA concentrations, therefore the red shift with the longer resonance wavelength was demonstrated.

After the molecular self-assembly of thiol molecules on the metal surface, the monolayer formation is driven by a strong coordination of sulfur with the metal, accompanied by van der Waals interactive forces between the alkyl chains [11]. The reflective optical power was higher as the MTB DNA concentration was lower, which was the opposite to the non-immobilization situation due to the self-assembled monolayer structure.

Compared with the previous publication for avian influenza-DNA hybridization detection from the visible wavelength scanning [17], the sensitivity was around 1 nm/μM. In this paper, the sensitivity was 1.04 nm/(μg/mL) using 5′-thiol end labeled DNA probes, which can be coverted to 3.26 nm/μM considering the 3313 base pairs of the MTB DNA (base pair = 700 g/mole), and demonstrated more sensitivity. Moreover, the HP 81640A tunable laser source we were using provided very slow wavelength scanning, so the observed response time was not as good as in the previous publication [2].

The piezoelectric TB DNA-based biosensor technology developed without PCR amplification showed a lowest concentration of 0.25 μM [3]. The highest sensitivity and resolution we could achieve for MTB DNA were 1.04 nm/(μg/mL) and 0.9 ng/mL (=0.28 nM), respectively. The 0.2 μg/mL (=0.06 μM) MTB DNA concentration increase observed in this paper was better than that of the biosensor without PCR amplification [3], but was less sensitive than the variable-number tandem repeats-based PCR technology [5].

## 5. Conclusions

MTB DNA with 5′-thiol end labeled DNA probes was successfully demonstrated by the high sensitive telecommunication wavelength modulation on surface plasmon resonator because the telecommunication wavelength possesses high precision and stability control in optical fiber communications. The experimental data showed that the SPR sensitivity increased more than 13 times with the wavelength modulation after immobilization. The highest sensitivity and resolution for *MTB* DNA were 1.04 nm/(μg/mL) and 0.9 ng/mL, respectively, after the 0.001 nm high telecommunication wavelength resolution of TLS was applied. For higher sensing limit applications, the TLS built with a PM fiber could be utilized for significant improvement.
